# Activation of dopamine D2 receptors attenuates neuroinflammation and ameliorates the memory impairment induced by rapid eye movement sleep deprivation in a murine model

**DOI:** 10.3389/fnins.2022.988167

**Published:** 2022-10-05

**Authors:** Perla Ugalde-Muñiz, María Guadalupe Hernández-Luna, Stephany García-Velasco, Rafael Lugo-Huitrón, Jimena Murcia-Ramírez, Ricardo Jesus Martínez-Tapia, Roxana Noriega-Navarro, Luz Navarro

**Affiliations:** ^1^Laboratory of Neuroendocrinology, Departamento de Fisiología, Facultad de Medicina, Universidad Nacional Autónoma de México, México City, Mexico; ^2^Laboratory of Behavioral Neurobiology, Departamento de Fisiología, Facultad de Medicina, Universidad Nacional Autónoma de México, México City, Mexico

**Keywords:** neuroinflammation, REM sleep deprivation, dopamine, D2 dopamine receptor, memory

## Abstract

The proinflammatory state, which may be induced by sleep deprivation, seems to be a determining factor in the development of neurodegenerative processes. Investigations of mechanisms that help to mitigate the inflammatory effects of sleep disorders are important. A new proposal involves the neurotransmitter dopamine, which may modulate the progression of the immune response by activating receptors expressed on immune cells. This study aimed to determine whether dopamine D2 receptor (D2DR) activation attenuates the proinflammatory response derived from rapid eye movement (REM) sleep deprivation in mice. REM sleep deprivation (RSD) was induced in 2-month-old male CD1 mice using the multiple platform model for three consecutive days; during this period, the D2DR receptor agonist quinpirole (QUIN) was administered (2 mg/kg/day i.p.). Proinflammatory cytokine levels were assessed in serum and homogenates of the brain cortex, hippocampus, and striatum using ELISAs. Long-term memory deficits were identified using the Morris water maze (MWM) and novel object recognition (NOR) tests. Animals were trained until learning criteria were achieved; then, they were subjected to RSD and treated with QUIN for 3 days. Memory evocation was determined afterward. Moreover, we found RSD induced anhedonia, as measured by the sucrose consumption test, which is commonly related to the dopaminergic system. Our data revealed increased levels of proinflammatory cytokines (TNFα and IL-1β) in both the hippocampus and serum from RSD mice. However, QUIN attenuated the increased levels of these cytokines. Furthermore, RSD caused a long-term memory evocation deficit in both the MWM and NOR tests. In contrast, QUIN coadministration during the RSD period significantly improved the performance of the animals. On the other hand, QUIN prevented the anhedonic condition induced by RSD. Based on our results, D2DR receptor activation protects against memory impairment induced by disturbed REM sleep by inhibiting neuroinflammation.

## Introduction

Sleep seems to provide an evolutionary advantage to organisms; it plays a key role in energy balance, immune enhancement, and reorganization of neuronal circuits and facilitates the elimination of waste from the brain (Krueger et al., 2016; Cao et al., 2020). Nevertheless, sleep function remains controversial. A widely used approach to analyze sleep functions has been to extend a subject’s wakefulness, that is, to deprive him/her of sleep. This deprivation can be total or selective, such as rapid eye movement (REM) sleep deprivation (RSD) (Porkka-Heiskanen et al., 2013; Kushida, 2016).

In this sense, memory impairments are some of the better-known events derived from low-quality sleep or sleep deprivation (SD) in both humans and rodents (Walker, 2008; Killgore, 2010; McCoy and Strecker, 2011). For example, chronic SD induces impairments in spatial memory performance, a function dependent on hippocampus integrity (McCoy et al., 2013; Ward et al., 2017). Moreover, this may be related to low concentrations of the brain-derived neurotrophic factor (BDNF) in the hippocampus, an important neurotrophin involved in cognitive functions and synaptic plasticity (Zielinski et al., 2014). Also, intracellular signaling related to these processes is affected, such as the cAMP pathway (Abel et al., 2013).

The behavioral manifestations are diverse and depend on the context of the SD; indeed, these are not restricted to cognition; anxiety and depression-related behaviors have also been described in mice after SD (Daniele et al., 2017; Moon et al., 2018). Similar effects were observed in healthy human adults who exhibited an anxiety condition accompanied with some degree of depression after acute SD (Babson et al., 2010). In this context, neuroinflammation could be a significant factor contributing to the detrimental effects of SD on cognition and mood alterations. RSD-induced learning and memory impairments are often accompanied by an inflammatory response (Piber, 2021), mainly by increases in brain proinflammatory cytokines and microglial activation, where deficits in spatial memory are manifested (Wadhwa et al., 2017). Further, both acute and chronic SD cause alterations in the immune response characterized by the presence of increased levels of proinflammatory mediators, such as tumor necrosis factor-α (TNF-α), interleukin-1β (IL-1β), IL-6, IL-17A, and the C-reactive protein (CRP) (Hurtado-Alvarado et al., 2016), and other inflammatory molecules such as cyclooxygenase-2 (COX-2), inducible nitric oxide synthase (iNOS), endothelin-1 (ET-1), vascular endothelial growth factor (VEGF), and insulin-like growth factor 1 (IGF-1) (Zager et al., 2007; Ibrahim et al., 2011; Ruiz et al., 2012; Porkka-Heiskanen et al., 2013; He et al., 2014; Hurtado-Alvarado et al., 2016).

Recent evidence has shown that 96 h of RSD enhances the inflammatory response in the hippocampus by increasing IL-1β and IL-6 levels from the first-day post of SD, accompanied by activated astrocytes in CA1 (Hou et al., 2019). Even 24 h of RSD raises IL-1 expression in the liver and brain (hippocampus, hypothalamus, and prefrontal cortex); similarly, TNF-α expression was upregulated in the liver, spleen, and prefrontal cortex among mice exposed to RSD vs. controls (Ashley et al., 2016).

Dopamine (DA), a catecholamine neurotransmitter, is derived from L-tyrosine, which is converted to L-Dopa by the enzyme tyrosine hydroxylase (TH), primarily in dopaminergic neurons. Once DA is synthesized, it is stored in synaptic vesicles for future release (Matt and Gaskill, 2020). On the other hand, several research groups have shown that astrocytes and microglia can also synthesize and metabolize DA. Likewise, other immune cells such as macrophages, T-lymphocytes, B-lymphocytes, and dendritic cells have the necessary elements to synthesize DA (Pinoli et al., 2017; Thomas Broome et al., 2020; Li et al., 2022). DA modulates immune responses by activating dopamine receptors (DRs), members of the G protein-coupled receptor (GPCR) superfamily. Dopamine receptors are divided into two subgroups, D1-like (D1 and D5) and D2-like (D2, D3, and D4) (Matt and Gaskill, 2020). Both groups have been widely expressed in neurons and several immune cell populations, for example, in mouse NK, T cells, macrophages, and dendritic cells (Sarkar et al., 2010; Mikulak et al., 2014). In activated macrophages, DA increases interferon-gamma (IFNγ) concentrations and thus indirectly induces their phagocytic activity (Ali et al., 1994; Roy and Rai, 2004). Moreover, Haskó showed that dopamine D2 receptor (D2DR) activation with specific agonists, like bromocriptine and quinpirole (QUIN), decreases TNF-α and nitric oxide release from macrophages after exposure to lipopolysaccharide (LPS) (Haskó et al., 1996). Microglial cells, the resident macrophages in the central nervous system (CNS), and the main cells responsible for immune surveillance in the brain express both D1DR and D2DR. Several molecular events occur in the microglial response during inflammatory stimuli. Recent evidence identifies the dopaminergic system as a critical factor since microglial cells exhibit LPS-induced overexpression of D1DR and D4DR *in vitro* (Thomas Broome et al., 2020). Furthermore, the binding of DA to microglial D1DR positively regulates the synthesis of nitric oxide (Chang and Liu, 2000; Färber et al., 2005). DA regulates proinflammatory responses in microglia stimulated with LPS by modulating ERK1/2 and NF-κB signaling, which play a role in DA-mediated suppression of nitric oxide generation by altering iNOS transcription (Wang et al., 2019).

Noticeably, the entire landscape of DA activities is far from being elucidated, and the comprehension of the D2DR is even more limited than other dopaminergic receptors, additionally, albeit deleterious effects of SD on behavioral and neuroinflammatory events have been explored, little is known about the participation of D2DRs in the context of neuroinflammation derived from SD and its effects on cognition and other behavioral manifestations. This work characterized the proinflammatory state induced by RSD in CD1 mice. In parallel, deficits in long-term memory retrieval and anhedonia behavior were evidenced in the animals. Subsequently, we tested whether the activation of D2DR, using QUIN, ameliorates the cognitive impairment and the anhedonia behavior by potentially an anti-inflammatory effect. Thus, the D2DR seems to be an attractive target to elucidate the neurochemical mechanisms regulating inflammation behavior in sleep homeostasis.

## Materials and methods

### Experimental animals

All experiments were performed with 8-week-old male CD1 (ICR) mice maintained under standard conditions on a 12:12 h light-dark cycle (lights on and off at 7:00 a.m. and 7:00 p.m., respectively), with water and food provided *ad libitum*. Animal handling and experimentation strictly followed the Guidelines for Care and Use of Laboratory Animals published by the National Institutes of Health and the Guidelines of the Mexican Law of Animal Protection for the Use and Care of Laboratory Animals (Norma Oficial Mexicana NOM-062-ZOO-1999). All experimental procedures were approved by the research and ethics committees of the Facultad de Medicina, Universidad Nacional Autónoma de México (CICUAL 016-CIC-2019).

### Experimental groups

**FIGURE 1:**
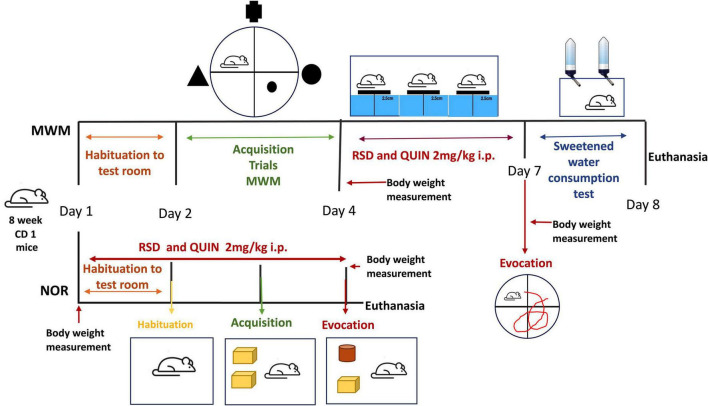
Schematic representations of REM-sleep deprivation (RSD) QUIN administration doses, regimens, and temporal strategies of behavioral tasks. The mice were subjected to 72 h of RSD with or without QUIN administration. The two behavioral tasks are indicated. For the Morris water maze (MWM), RSD was administered after acquisition and before the evocation test.

Mice were randomly divided into four groups: (1) a control group, (2) a REM sleep deprivation (RSD) group, (3) a QUIN group, and (4) an RSD + QUIN group. After the behavioral tests, mice were euthanized, and the serum and brain tissues were collected for ELISAs. The QUIN dose was 2 mg/kg/day (one i.p. injection/day) and was administered for 3 days 1 h prior to the RSD session. The control and RSD groups received saline solution injections. The body weight of the mice was measured before and after RSD and/or QUIN administration (Figure 1).

### Induction of rapid eye movement sleep deprivation

The multiple platform method was employed to prevent REM. This method is widely accepted to induce REM sleep disturbances (Ashley et al., 2016; Kincheski et al., 2017; Yin et al., 2017; Hou et al., 2019; Xue et al., 2019; Suresh et al., 2021). Briefly, six narrow platforms (8.5 cm in height and 2.5 cm in diameter) were placed (8–10 cm apart) inside an acrylic box filled with water (24°C) up to 3 cm from the top of the platforms. This arrangement allows animals to maintain social interaction and free movement from one platform to another, substantially reducing stress by immobilization or social isolation (Suchecki et al., 1998; Machado et al., 2004; Colavito et al., 2013). Access to water and food was available across the entire restriction period. Food pellets and water bottles were placed on a flat grid above the box during the experiment (approximately 6 cm above the platform’s surface). Six water bottles were strategically placed on the flat grid, ensuring each animal could reach the water. The tank was maintained on a 12:12 h light/dark cycle (lights on and off at 7:00 a.m. and 7:00 p.m., respectively). Four mice were handled for each experiment, so mice could easily move between the platforms but could not stretch across any two platforms to sleep. Thus, the animals awoke when they experienced REM sleep-induced atonia by touching the water. Sleep deprivation began at 12:00 p.m., and 24 h later, the mice were taken out of the deprivation box, injected with saline solution or QUIN, placed in their home cages, and given free access to food and water for 1 h. Sleep deprivation lasted 72 h. The tank was cleaned, and the water was replaced every day.

Mice treated with or without QUIN were subjected to RSD employing the modified inverted flowerpot method (multiple platform method), which is used to prevent REM sleep without affecting the non-REM (NREM) sleep (Lee et al., 2009). The animals from the control group stayed in their home cage.

### Cytokine titration

The levels of TNF-α, IL-6, IL-10, and IL-1β were analyzed using sandwich ELISAs according to the manufacturer’s instructions. Serum and brain tissues (hippocampus, cerebral cortex, and striatum) retrieved from mice trained and evoked in novel object recognition (NOR) test were used for cytokine determinations. After behavioral tests, mice were euthanized with an injection of sodium pentobarbital (50 mg/kg). Blood sampling was performed by cardiac puncture, and after clotting, blood was centrifuged to isolate the serum (12,000 rpm 15 min at 4°C). The hippocampus, cerebral cortex, and striatum were removed and homogenized in 500 μL of ice-cold lysis buffer (20 mM Tris, 0.25 M sucrose, 2 mM EDTA, 10 mM EGTA, and 1% Triton X-100) containing a cocktail of protease inhibitors.

The levels of all cytokines in serum and brain tissues were detected using specific ELISA kits from PeproTech (IL-6, 900-K50; IL-10, 900-K53; TNF-α, 900-K54; and IL-1β 900-K47) according to the manufacturer’s recommendations. All serum samples and brain tissue homogenates were incubated for 18 h at 4°C. Bound detection antibodies were detected using ABTS as substrate. Single optical density readings were recorded at 405 nm. All assays were performed in duplicate, and the sensitivities were 32–4,000 pg/mL for IL-1β, 16–2,000 pg/mL for IL-6, 30 pg/mL for IL-10, 32–2,000 pg/mL, and 8 pg/mL for TNF-α. At least four animals from each group were used for the statistical analysis.

### Morris water maze test

The spatial learning and memory of mice were assessed to evaluate cognitive performance. The mice from each group were placed in a pool filled with water (90 cm diameter; 19–21°C) and allowed to navigate the pool and reach a submerged escape platform (8 cm diameter, 0.3 cm under black-stained water). Three visual cues were placed 15 cm outside the pool, consisting of a triangle, a circle, and a cross (30 cm each) visible to the animals. During training sessions, mice navigated the pool until they found the platform. If they failed to locate the platform within 60 s, animals were gently guided to it and allowed to stay on it for 20 s. The task was considered learned when the average navigation time was less than or equal to 20 s in 4 different training sessions on 1 day. During the acquisition period, training started at 9:00 a.m. Once the last training was completed, animals were injected with QUIN and left in their home cages for one h, then placed in the RSD setup starting at 12:00 p.m. At 12:00 p.m. of the next day, the RSD session ended; mice were injected and left to recover for one h before starting the next RSD session, the same schedule as the last day. After completing 72 h of RSD, the mice were allowed to recover for one h in their home cage. The spatial long-term memory evocation test consisted in placing the animals in the pool lacking the platform and allowing them to navigate for 60 s; this was carried out at 1:00 p.m. Platform placement was randomly assigned through the four quadrants for each mouse to discard mouse preference/aversion for any quadrant. The latencies to reach the escape platform were analyzed for the acquisition period to evidence that every animal learned the task. Every animal test was video recorded using a web camera (Logitech), and every video obtained from the evocation test was analyzed using the open-source software ImageJ and the Animal Tracker application (Gulyás et al., 2016). The software allows the analysis of the video files post-test. The total time spent in the target and opposite quadrant, the number of crossings in the platform site, latencies to the platform zone, and the average distance to the platform site during navigation were estimated and analyzed for the evocation test. Moreover, the average swimming speed of animals was also evaluated.

### Novel object recognition test

Object acquisition and memory evocation were assessed in an arena with the walls and floor painted black (30 cm × 30 cm × 40 cm) (Reger et al., 2009) designed for this purpose. During the 3 days of the test, the mice underwent RSD and/or QUIN administration (Figure 1). The arena was kept in a separate room to eliminate the effects of visual and auditory distractions on the test. Washable plastic toys (mega blocks) and Falcon tubes that were different in color and shape but similar in size (approximately 10 cm × 8 cm) were selected as objects (Figure 5D). Novel and familiar object pairs were randomly assigned, and their positions were counterbalanced to further reduce the possible effects of the object or place preference/aversion. Objects were secured at opposite corners of the arena (6 cm from the wall) to ensure that a mouse could not displace them. Test sessions were recorded using an overhead camera. The arena and objects were cleaned with 70% ethanol to eliminate olfactory cues between sessions and animals.

The object recognition task was performed by dividing testing into 3 phases: habituation, acquisition, and recognition; each of the three phases started at 1:00 p.m. when ended each RSD session (see Figure 1), QUIN was administered, and mice were allowed to stay in their home cage one h before to be tested in the NOR arena. On day 1, QUIN was administered at 11:00 a.m. The next day, at 12:00 p.m., the mice received the second dose of QUIN, left in their home cage for one h, trained or tested according to the stage of the NOR protocol, and returned to the RSD setup. For habituation, each mouse was removed from its home cage, placed in the middle of the open arena, and allowed to explore for 5 min (Lueptow, 2017). The acquisition phase was performed after 24 h by placing a mouse into the arena with two identical sample objects for up to 10 min to allow for a criterion level of exploration and familiarization with the objects. Mice were tested with a 24 h intertrial interval. For the recognition task, one of the familiar objects was substituted with an unfamiliar object (Falcon tube, 50 ml). The time spent investigating objects was assessed manually from video recordings. Object investigation was defined as a mouse directing the nose toward, touching, or sniffing within 1 cm of the object but not standing, sitting, or chewing on the object. Time spent attending to objects per investigation during the acquisition trial was used to measure of object-based attention. The recognition index (RI) was calculated by dividing the time spent exploring the novel object (TNO) by the total time spent exploring both objects (TE):

Recognition index = TNO/TE

### Sucrose consumption preference test

Rodents naturally prefer to drink sweetened water when given a two-bottle free-choice regimen with access to sucrose solution and regular water (Willner et al., 1987; Sobrian et al., 2003; Goshen et al., 2008; Zhou et al., 2011; Liu et al., 2018). However, when mice are “depressed,” they fail to select the sweetened water, a phenomenon known as anhedonia, the lack of feeling pleasure. Many studies suggest that 1–2% (wt/vol) sucrose solution is the optimal concentration for offering rodents alongside water to distinguish whether mice or rats are “depressed” or not (Liu et al., 2018). We evaluated sucrose consumption preference to measure anhedonia as indicator of depression-like behavior.

The animals included in the anhedonia test were the same trained and evoked in the Morris water maze (MWM). The last RSD session ended at 12 p.m., mice were allowed to stay in their home cages for one h, were evoked and consumption tests began at 2:00 p.m. Each mouse was individually placed in a measurement cage for 24 h at the end of the last deprivation session. Two graduated drinking tubes (falcon 50 ml) were presented, one containing tap water and the other with 2% sucrose solution. The animals had free access to food. The volume consumption was determined in milliliters, and sucrose preference was calculated as the percent of sucrose consumption out of the total drinking volume. Sucrose preference was calculated by the following formula: Preference = (Sucrose intake/Total intake) × 100. A percent consumption of less than 65% was considered anhedonia (Bessa et al., 2009). At the end of the test, animals were returned to group housing with free access to food and water.

### Statistical analysis

GraphPad Prism 9 software was used for statistical analyses of all data. Data are reported as the mean ± standard error of the mean (SEM). Analysis of evocation parameters in MWM and cytokine levels was carried out using one-way ANOVA and Tukey as a *post-hoc* test. The recognition index and exploration time data in NOR were analyzed by unpaired Student’s *t*-test; *p* < 0.05 was considered statistically significant.

## Results

### Effect of quinpirole on rapid eye movement sleep deprivation-induced neuroinflammation

**FIGURE 2:**
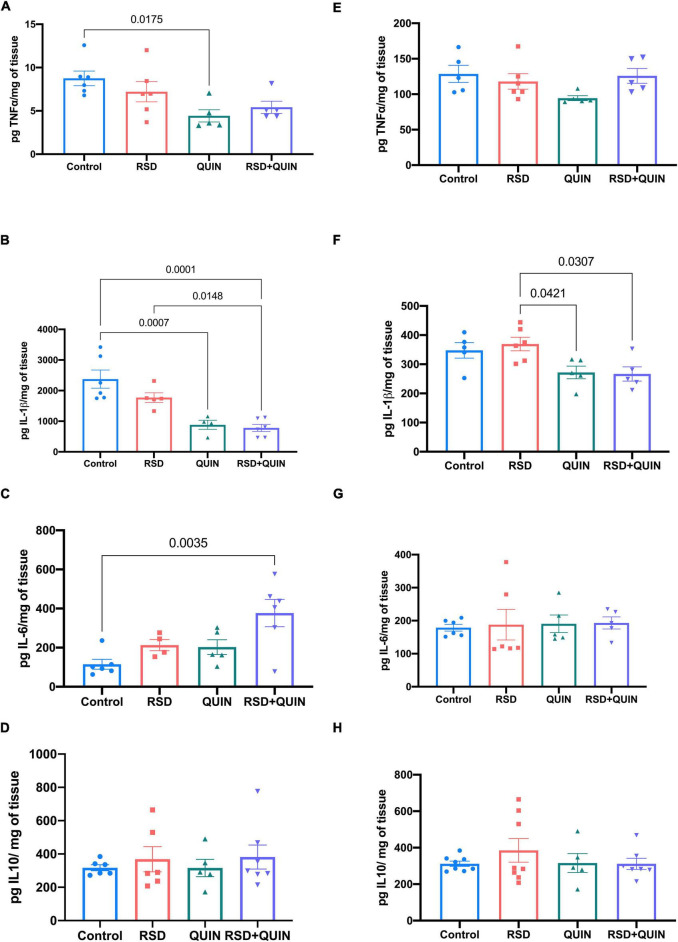
Effect of Quinpirole (QUIN) administration on REM sleep deprivation (RSD) mice on cytokine levels in the cerebral cortex and striatum. Mice were administered with QUIN (2 mg/kg/day for 3 days) and subjected to RSD. The levels of cytokines were analyzed using ELISAs in the striatum **(A–D)** and the cerebral cortex **(E–H)**. Data are presented as the mean ± SEM and were analyzed using one-way ANOVA, followed by Tukey’s *post-hoc* test. For striatum TNF-α *n* = 5–6, IL-1β *n* = 5–6, IL-6 *n* = 5–6, and IL-10 *n* = 5–7. For cerebral cortex TNF-α *n* = 5, IL-1β *n* = 5, IL-6 *n* = 5–6, and IL-10 *n* = 5–8. Significant differences among the experimental groups are indicated.

We evaluated three different brain regions (cerebral cortex, hippocampus, and striatum) and serum to determine whether RSD triggers an inflammatory state alongside the effect of QUIN administration. The ELISAs tests of the cytokines IL-6, IL-1β, IL-10, and TNF-α revealed differential immune responses within the tissues. In the cerebral cortex, levels of the TNF-α, IL-1β, IL-6, and IL-10 proteins did not increase significantly after RSD (Figures 2E–H) however, the cortex obtained from RSD mice and treated with QUIN showed decreased levels of IL-1β as compared to RSD mice (*p* < 0.0307) (Figure 2F).

Furthermore, in the striatum, RSD did not induce changes in the levels of any cytokine (Figures 2A–D), but the simple administration of QUIN to control mice induced a decrease in TNF-α (*p* < 0.0175; Figure 2A) and IL-1β (*p* < 0.0007) (Figure 2B). However, the RSD + QUIN group showed increased levels of IL-6 compared to the control group (*p* < 0.0035) (Figure 2C).

**FIGURE 3:**
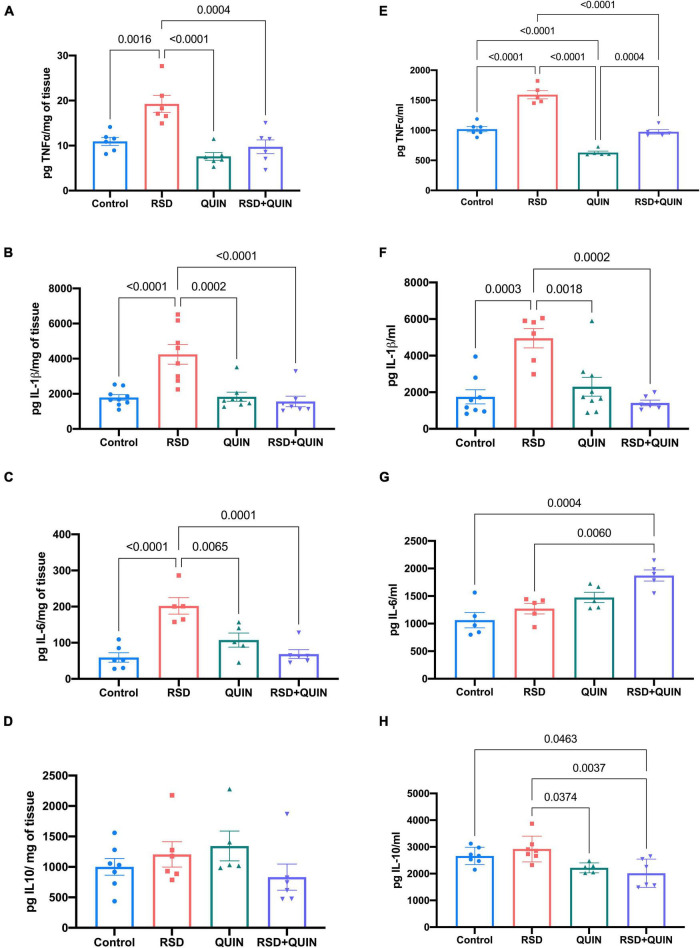
Quinpirole (QUIN) attenuated the increases in the levels of proinflammatory cytokines evoked by REM sleep deprivation (RSD). Effect of QUIN on hippocampal and serum cytokine levels in RSD mice. Mice were administered QUIN (2 mg/kg/day for 3 days) and subjected to RSD. The levels of TNF-α **(A)**, IL1β **(B)**, IL6 **(C)**, and IL10 **(D)** in the hippocampus and TNF-α **(E)**, IL1β **(F)**, IL-6 **(G)**, and IL-10 **(H)** in serum were analyzed by ELISA. Data are presented as means ± SEM and were analyzed using one-way ANOVA, followed by Tukey’s *post-hoc* test. For hippocampus TNF-α *n* = 6, IL-1β *n* = 7–9, IL-6 *n* = 5–6, and IL-10 *n* = 5–7, for serum TNF-α *n* = 5–6, IL-1β *n* = 6–8, IL-6 *n* = 5, and IL-10 *n* = 5–7. Significant differences among the experimental groups are indicated.

In the hippocampus, RSD increased the levels of TNF-α (*p* = 0.0016) (Figure 3A), IL1β (*p* < 0.0001) (Figure 3B), and IL-6 (*p* < 0.0001) (Figure 3C). Mice subjected to RSD and administered with QUIN showed decreased levels of TNF-α (*p* = 0.0004) (Figure 3A), IL-1β (*p* < 0.0001) (Figure 3B), and IL-6 (*p* = 0.0001) (Figure 3C) as compared to RSD mice, and no significant changes were observed in the levels of the anti-inflammatory cytokine IL-10 (Figure 3D). A similar tendency in serum was observed, where RSD induced an increase in the levels of TNF-α (*p* < 0.0001) (Figure 3E) and IL-1β (*p* = 0.0003) (Figure 3F) compared to the control group (Figure 3G). In contrast, QUIN administration significantly reduced this increase in TNF-α (*p* < 0.0001) levels (Figure 3E) and IL-1β (*p* = 0.0002) (Figure 3F). Surprisingly, the two groups that received QUIN showed a decrease in the IL-10 levels, QUIN (*p* = 0.0374) and RSD + QUIN (*p* = 0.0037) (Figure 3H), compared to the RSD group. These observations confirm that 72 h of RSD is sufficient to induce inflammation. Under our settings, both IL-1β and TNF-α increased systemically and in the brain, albeit not every brain area responded equally. On the other hand, and as expected, the D2DR activation using QUIN attenuated the neuroinflammation evoked by RSD, confirming the immunomodulatory effect of this receptor.

### Quinpirole ameliorates the rapid eye movement sleep deprivation-induced episodic memory impairment

The experimental strategy followed here allowed us to evaluate the effects of RSD-derived neuroinflammation on behavior, we focused on determining the integrity of cognitive functions by evaluating long-term memories in two different tasks, MWM and NOR. In our experiments, no effect was observed on the learning stage in the MWM task (data not shown) when mice were sleep-deprived within training days. Thus, we aimed to evaluate whether memory retrieval was impacted.

**FIGURE 4:**
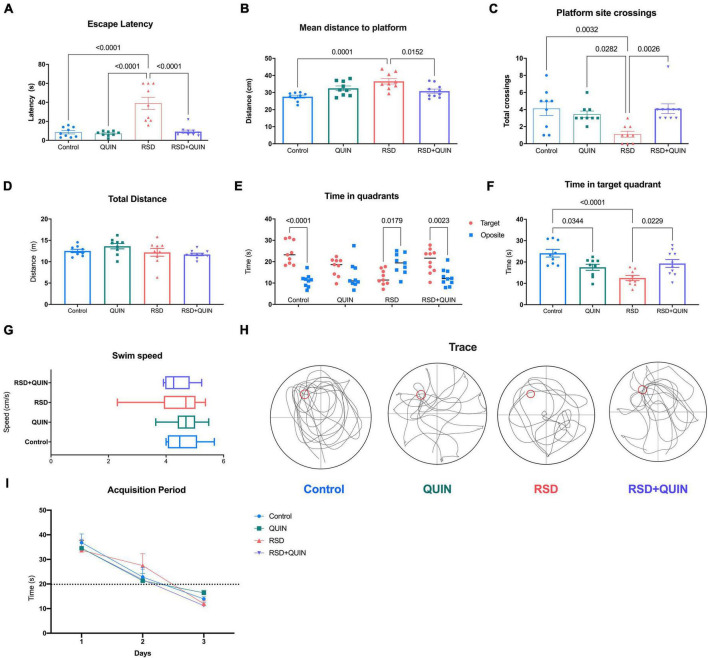
Effect of Quinpirole (QUIN) and REM sleep deprivation (RSD) on spatial memory. The RSD was administered for 72 h starting after the last training session in the MWM. The evocation test analysis **(A–H)** consisted of 60 s of navigation assessed at the end of the RSD. The following evocation parameters are shown escape latency to the site of the platform **(A)**, average navigation distance to the platform **(B)**, the number of platform site crosses **(C)**, total distance navigated **(D)**, total time spent in target and opposite quadrants **(E)**, time spent in the target quadrant **(F)**, navigation speed **(G)** and typical navigation traces **(H)**. Repeated training sessions over 3 days resulted in shorter latencies and higher performance in platform localization **(I)**. Mice were administered with QUIN (2 mg/kg for 3 days) and subjected to RSD after acquisition. Data are presented as the mean ± SEM and were analyzed using one-way ANOVA followed by Tukey’s *post-hoc* test **(A–D,F,G)** and Student’s *t*-test **(E)**. Control *n* = 9, QUIN *n* = 9, RSD *n* = 9, RSD + QUIN *n* = 10. Statistically significant differences among the experimental groups are indicated.

The MWM test was conducted after every animal learned and consolidated the task. Our data showed that RSD for three consecutive days led to a spatial memory evocation deficit in mice, even when the animals had already learned the task (Figure 4I). As shown in Figure 4A, compared with the control group, the escape latencies of mice in evocation tests were significantly higher for the RSD group (*p* < 0.0001). Additionally, these animals navigated significantly farther from the platform during the test (Figure 4B) (*p* < 0.0001). Furthermore, RSD mice failed to locate the platform site (Figure 4C) (*p* = 0.0032); and the time spent navigating the target quadrant by RSD mice was significantly lower than that of the control group (*p* < 0.0001) (Figure 4F).

Quinpirole administration throughout the 3 days of RSD and after the training period noticeably reversed the cognitive impairment caused by RSD. QUIN administration to RSD mice decreased the escape latencies (Figure 4A) (*p* < 0.0001), shortened the average distance traveled to the platform (Figure 4B) (*p* = 0.0152), increased the number of crosses of the platform site (Figure 4C) (*p* = 0.0026) and increased the time spent in the target quadrant vs. the opposite quadrant (Figure 4E) (*p* = 0.0023) as the total time spent in the target quadrant (Figure 4F) (*p* = 0.0229).

Surprisingly, mice treated with QUIN alone spent less time in the target quadrant than the control group (*p* = 0.0344; Figure 4F). However, no differences were observed in the total distance traveled (Figure 4D) or navigation speed (Figure 4G) for any experimental group. Representative memory traces are shown for every group (Figure 4H); RSD mice traveled a more complex path than control mice, and QUIN resulted in a trace comparable to that of a control animal. These data strongly suggest that RSD mice failed to evoke spatial memory from a previously learned task; however, QUIN reduced this impairment, and this effect seemed to be independent of the locomotor ability.

Subsequently, we were interested in testing if RSD induced deterioration in another memory task, such as the NOR test, and if QUIN could reverse this deterioration.

**FIGURE 5:**
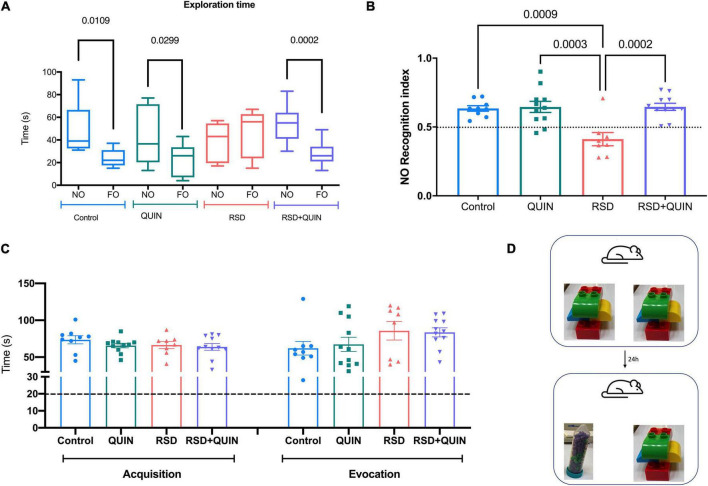
Rapid eye movement (REM) sleep deprivation (RSD) for 72 h impaired recognition memory performance, and Quinpirole (QUIN) attenuated this effect. Data are shown as the **(A)** novel and familiar object exploration time, **(B)** Recognition index of the Novel object recognition test (NOR-RI), **(C)** total exploration times of both objects in acquisition and evocation, and **(D)** objects used in the acquisition and evocation test. Mice were administered QUIN (2 mg/kg for 3 days) and subjected to RSD. Data are presented as the mean ± SEM and were analyzed using one-way ANOVA, followed by Tukey’s *post-hoc* test **(B,C)** and Student’s *t*-test **(A)**. Control *n* = 9, QUIN *n* = 11, RSD *n* = 8, RSD + QUIN *n* = 11. Significant differences among the experimental groups are indicated.

Object recognition evocation was tested after 72 h of RSD and/or QUIN administration. In the evocation test, rodents spent more time exploring a novel object (NO) over a familiar object (FO). In our settings, only the animals subjected to RSD failed to recognize the NO (Figure 5A). We observed that RSD negatively affected NO preference (control RI = 0.63 vs. RSD RI = 0.40; *p* = 0.0009) (Figure 5B). These data suggest that long-term recognition memory was impaired in RSD mice, as shown by a reduced preference to investigate novel objects during the object recognition phase of the task (Figure 5B). However, QUIN administration reversed the impairment produced by RSD (RSD RI = 0.40 vs. RSD + QUIN RI = 0.64; *p* = 0.0002) (Figure 5B).

The total exploration times were compared between experimental groups in the acquisition or the evocation phase to confirm that RSD did not affect the exploratory behavior of mice (hence, attention to the task). No statistical differences were observed in acquisition or evocation comparing the four groups, indicating that animals explore equally the objects (Figure 5C). In the NOR test, it is proposed that animals should reach a minimum of 20 s of exploration in both phases to consider they are executing the task correctly. All animals from all experimental groups performed the task accomplishing this criterion, even the RSD group; thus, we can assume that exploration behavior was not altered (Lueptow, 2017).

The data obtained from both memory tasks prove that the neuroinflammation derived from RSD negatively impacts memory retrieval in two different tasks, indicating a strong relationship between proinflammatory cytokines and cognitive functions in the RSD. In concordance, spatial memory impairment is likely due to the hippocampus’s neuroinflammation. Interestingly, our settings allowed us to distinguish that such damage reflects on memory recall. These data also show that activation of D2DR prevented the cognitive deficit, similar to that observed in ELISAs results where it had anti-inflammatory effects.

### Quinpirole ameliorates anhedonia-like behavior and prevents loss of body weight induced by rapid eye movement sleep deprivation

**FIGURE 6:**
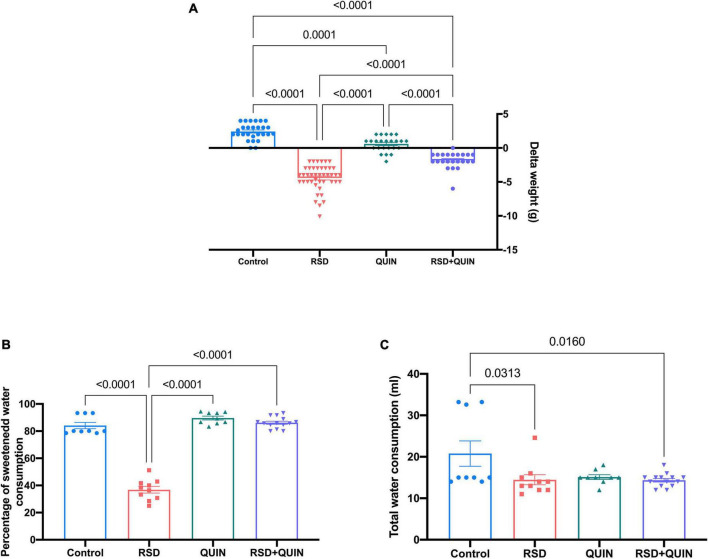
Body weight and sucrose water consumption decreased after REM sleep deprivation (RSD), whereas Quinpirole (QUIN) administration attenuated these changes. Mice were administered with QUIN (2 mg/kg for 3 days) and exposed to RSD. Delta of body weight **(A)**, sucrose water consumption **(B)**, and total water consumption **(C)**. Data are presented as the mean ± SEM and were analyzed using one-way ANOVA, followed by Tukey’s *post-hoc* test. Body weight measurements: Control *n* = 27, QUIN *n* = 24, RSD *n* = 43, RSD + QUIN *n* = 23. Sucrose water consumption: Control *n* = 9, QUIN *n* = 9, RSD *n* = 10, RSD + QUIN *n* = 14. Significant differences among the experimental groups are indicated.

We also measured the body weights before and after sleep deprivation and QUIN administration. Mice in the RSD group showed decreased body weight compared to the control group (*p* < 0.0001). On the other hand, QUIN administration attenuated the body weight loss induced by RSD (Figure 6A). Surprisingly, the administration of QUIN significantly reduced the weight of the mice compared to the control group.

Anhedonia-like behaviors are reflected by decreased sucrose water consumption since rodents have an innate preference for sweetened water over regular water. As shown in Figure 6B, RSD induced an anhedonia-like state; these mice consumed significantly less sucrose water than animals from the control group (*p* < 0.0001). On the other hand, the RSD + QUIN group showed an increased sucrose preference when compared to the RSD group (*p* < 0.0001) (Figure 6B). Animals from the control group, QUIN group, and RSD + QUIN group exhibited similar sucrose water preferences, indicating that QUIN *per se* does not influence motivational behaviors. Regarding the total consumption of liquids, we observed a significant difference between the control group and the RSD (*p* = 0.0313) and RSD + QUIN (*p* = 0.0160) groups (Figure 6C). Taken together, data from body weights, sucrose consumption, and cytokines analysis indicate that the D2DR stimulation prevents depression-related behaviors through likely modulation of immune response in the brain.

## Discussion

Sleep fragmentation has been extensively studied; consequences of sleep loss vary from metabolic parameters and mood alterations to cognitive impairments and increases in the risk of developing several diseases. For decades, the experimental manipulation of sleep stages (either REM or NREM) has clarified some of the mechanisms involved in such effects. In order to determine whether REM sleep deficiencies induce neuroinflammation in the present work, a modified flowerpot method was used to induce sleep deprivation, the multiple platforms method, which is well-accepted to induce REM sleep deprivation. Our data indicate that proinflammatory events are triggered when mice are deprived of REM sleep. Then, the deficit in memory retrieval and anhedonia behavior was also evidenced in these animals; most likely, the neuroinflammation underlies such effects. Nevertheless, under the experimental approaches presented here, we are unable to discard NREM unbalances since selective deprivation of one sleep stage invariably affects the other (Colavito et al., 2013).

On the other hand, our results evidenced the remarkable effect of QUIN administration in modulating the inflammatory response, attenuating cognitive deficits, and reducing the depression-like state in animals subjected to RSD. Thus, the activity of the D2DR seems to link the inflammation events with mnemonic functions and even motivation.

### Quinpirole attenuates neuroinflammation after rapid eye movement sleep deprivation

Cytokines are mostly related to the immune system and are released through either systemic or central responses. Nevertheless, many studies have reported that fluctuations in these molecules drive crucial changes in learning and memory (Bourgognon and Cavanagh, 2020). Specifically, IL-1β exerts a concentration-dependent effect on memory; when it was injected directly into the hippocampus after training, memory consolidation resulted in impaired contextual memory tasks (Gonzalez et al., 2009). In contrast, IL-1β seems to exert a dual effect on memory tasks; the expression of its messenger RNA (mRNA) is induced in the hippocampus after the Y-maze test (Labrousse et al., 2009), and intracerebroventricular injections of a low dose of this cytokine influence memory in a dose-dependent manner (Goshen et al., 2007). Our results revealed a two-fold increase in IL-1β and TNF-α levels in RSD mice, suggesting synergism in the hippocampus, thus inducing memory impairments. Some reports show that TNF-α is a pivotal regulator of even more complex pathways. Its action depends on the brain region and the inflammatory stimulus that triggers its release (Shin et al., 2014; Bourgognon and Cavanagh, 2020). However, the inhibition of long-term potentiation (LTP) (thus provoking memory deficits) prevails as a key feature of this cytokine (Butler et al., 2004; Singh et al., 2019).

Anti-inflammatory actions for D2 receptors have been explored in the systemic immune system (Sarkar et al., 2010) and neuroinflammatory models (Peiser et al., 2005; Zhang et al., 2015; Liu et al., 2021); however, their role in sleep deprivation models is poorly understood. Here, we revealed the potential of QUIN in attenuating neuroinflammation after RSD, since its administration decreased canonical proinflammatory cytokines levels (IL-1β and TNF-α) in the brain and serum. Interestingly, the inflammatory response was region-dependent since RSD raised the levels of both cytokines in the hippocampus; however, this effect was not observed in the striatum and the cerebral cortex.

Additionally, QUIN prevented the RSD-induced increase in inflammatory cytokine levels in the hippocampus. Previous studies have shown that QUIN decreases IL-1β and MCP-1 levels by suppressing the CRYAB/NF-kB inflammatory pathway in an intracerebral hemorrhage model, and this change was associated with reduced microglial activation (Zhang et al., 2015). Furthermore, another study showed that QUIN administration reduces secondary glial cell activation and neuroinflammation after traumatic brain injury by regulating the Akt/GSK3-β signaling pathway (Alam et al., 2021). Both reports stated that the immunomodulatory effect of QUIN is mediated by microglia; notably, in our work, the anti-inflammatory effect of QUIN was reproduced; however, the microglial state remains to be elucidated.

The anti-inflammatory effect of D2DR has been reported previously. Activation of D2-like receptors promotes the formation of phosphoprotein phosphatase 2 (PP2A), Akt, and β-arrestin2 protein complexes. β-arrestin2 acts as a G-protein-independent signaling mediator by scaffolding other proteins such as kinases and, in this way, regulate the Akt/NF-κB signaling pathway and inhibits the production of proinflammatory cytokines (Beaulieu et al., 2007; Li et al., 2022). Another study reported that D2DR negatively regulates the Akt/NF-κB inflammatory signaling pathway and exerts protective effects against LPS liver injury by reducing the serum aminotransferases activity and TNF-α production (Yue et al., 2021). Data presented here indicate that D2DR activation reduces the IL-1β, IL-6, and TNF-α production. Moreover, based on previous antecedents, the D2DR anti-inflammatory effect is likely due to Akt inactivation and the promotion of PP2a, Akt, and β-arrestin 2 complex. The consequent inhibition of the IκB/NF-κB signaling pathway causes a decrease in the release of cytokines, such as TNF-α and IL-1β (Li et al., 2022), suggesting that D2DR activation might serve as a therapeutic target for regulating neuroinflammation evoked by RSD.

Surprisingly, our results indicate that QUIN administration reduced the striatum levels of TNF-α and IL-1β compared to the control group (Figures 2A,B). Studies in brain D2DR expression patterns highlight that striatum is particularly enriched with this receptor (Charuchinda et al., 1987; Levey et al., 1993). The high density of D2DR in this region may be responsible for this effect, suggesting that D2DR activity regulates the inflammatory signaling even without a proinflammatory stimulus such as RSD. However, these mechanisms remain to be elucidated under the experimental approach used in this work.

Similar to the hippocampus, RSD induced a TNF-α and IL-1β increase in the serum of these mice (Figures 3E,F). These cytokines are produced by activating M1 macrophages, which play a key role in inflammatory response (Kadomoto et al., 2022). Another work addressed that DRD2 activation also exerts a protective effect against acute pancreatitis and reduces systemic inflammation by inhibiting M1 macrophages through the bone marrow-specific D2DR signaling control (Han et al., 2020). DRD2 activates PP2A, which inhibits the production of inflammatory factors by inhibiting the phosphorylation of IκBα and NF-κBp65. In this context, QUIN administration potentiates systemic levels of TNF-α and IL-1β (Figures 3E,F) via the activation of D2DR on peripheral macrophages, inhibiting NK-kB activation. A deeper analysis of this pathway would provide valuable data to lighten the conditions underlying the D2DR modulation of the immune response.

Additionally, future experiments will assess the contribution of glial cells in our model. A recent study reports that total sleep deprivation induces neuroinflammation and spatial memory decline, possibly derived from hyperactivated glial cells; interestingly, the pharmacological inhibition of microglia activation reverts the deleterious effects (Wadhwa et al., 2017). Our findings evidence that fragmentation of REM sleep is sufficient to induce neuroinflammation leading to memory deficits; however, little is known regarding the long-term effects of sub-chronic RSD on cytokine levels. Chronic sleep restriction (but not REM) for 30 days by the gentle touch method causes increased levels of IL-1β and IL-6. This restriction potentiates the effect of subtoxic infusion of amyloid-β oligomers in memory and neuroinflammation (Kincheski et al., 2017). The temporality of inflammatory response in our RSD setting remains to be elucidated.

### Memory deficits by rapid eye movement sleep deprivation are ameliorated by quinpirole

Dopamine receptors modulate memory formation through complex mechanisms; specifically, the D2DR activation seems to be crucial for hippocampus-dependent memories (Liggins, 2009). It strongly influences LTP and long-term depression (LTD), both processes involved in synaptic plasticity that subsequently regulates learning and memory (Rocchetti et al., 2015). In humans, functional MRI and PET analyses revealed that D2DR contributes to episodic memories in a hippocampus-dependent manner, highlighting the relevance of connectivity between this region and the caudate (Nyberg et al., 2016).

Sleep deprivation (either REM or NREM) affects learning and memory function. Its effect on cognitive deficits has been explored in humans (Walker, 2008; Killgore, 2010) and rodents, as reviewed by McCoy and Strecker (2011), stating the crucial role of quality sleep in mnemonic functions. In our settings, we aimed to disturb the REM stage, and this was induced for 72 h; similar approaches have been reported in rats subjected to RSD for 48 h or 96 h. Animals showed impaired memory retention of previous consolidated spatial learning (Li et al., 2009; Hou et al., 2019). The MWM paradigm has been proven to be the benchmark in spatial memory assessment. Its versatility and relatively easy setup allow the analysis of different stages in learning and memory (Vorhees and Williams, 2006). In this work, the RSD did not affect on learning period of the MWM (data not shown). However, animals exhibited poor memory retrieval when sleep-deprived after the acquisition period and before the evocation test (Figure 4). Recent evidence supports these observations since regulation and persistence of spatial memory involve the D2DR activity in the ventral pallidum within the basal ganglia when activated with QUIN (Péczely et al., 2016). As discussed above, the data obtained from ELISAs assays showed a robust increase in proinflammatory levels in the hippocampus; thus, the spatial memory deficit was expected, and it seems that local inflammation derived from RSD is responsible for such behavior.

Since QUIN induced significant recovery from spatial memory impairment caused by RSD, we were interested in testing an additional type of memory. The NOR memory task analyses the spontaneous exploring behavior of rodents in response to novelty in a familiar context (Bevins and Besheer, 2006; Broadbent et al., 2010; Antunes and Biala, 2012; Cohen and Stackman, 2015). The animals are not motivated or punished to resolve the NOR task; thus, the stimulation of animals relies on habituation and training with subsequent retrieval tests (Antunes and Biala, 2012). Some studies have also used the NOR task to evaluate attention; however, we aimed to test memory retrieval (Carey et al., 2008; Taglialatela et al., 2009). Recently, a study reported that D1DR activity regulates the synaptic plasticity in the dentate gyrus and significantly contributes to completing the NOR task in mice (Yang et al., 2017). Thus, we aimed to explore the effect of RSD on memory performance in the NOR test and whether QUIN administration relieved this effect. Results in the NOR test were similar to that observed on the MWM in our settings. Animals under RSD had poor novelty recognition (Figure 5B), and QUIN ameliorated the retrieval deficit in the task. The enhanced memory performance observed in both tasks may be explained by previous findings where D2DR activity increases the expression of BDNF and kidney and brain-expressed protein (KIBRA), enhancing the performance in episodic memories tasks (Péczely et al., 2016). The fact that QUIN ameliorates the deficient performance in both tasks after RSD denotes the relevance of D2DR in modulating these functions. However, based on our data, only activating this receptor has no effects on mnemonic capabilities, suggesting that the D2DR offers protection under an additional stimulus, here the inflammation induced by RSD.

A limitation of our work is that systemic administration of QUIN impedes the definition of the role of specific cell populations (e.g., microglia and systemic immune cells). Experiments generating KO mice targeting the D2DR in specific immune cells would solve this paradigm, on the other hand, to prove the relevance of D2DR activity and local inhibition of D2DR and compare its effect vs. the rescue observed with QUIN.

### Quinpirole prevented anhedonia-like behavior in rapid eye movement sleep deprivation

The weight loss in animals subjected to RSD was expected. However, mice from the QUIN group also exhibited body weight loss compared to the control group (Figure 6A). Previously it has been reported that QUIN administration (0.2 mg/kg, i.p.) reduces the food intake 24 h after administration (Kuo, 2002). This effect is associated with declines in leptin, indirectly inducing the excitability of dopaminergic neurons in the ventral tegmental area (VTA) and may contribute to a change in feeding behavior through the mesolimbic dopaminergic system (Murakami et al., 2018).

Anhedonia is a complex phenomenon of processing and responding to rewards indicating depression-like behaviors (Scheggi et al., 2018). We employed the sucrose consumption task in this study to evaluate whether animals show alterations in this system. Previous reports have provided evidence that 72 h of RSD induces depression-like behaviors; animals exhibit increased immobility latency in the forced swimming and tail suspension tests. Both alterations are mainly attributed to serotonin (5-HT) reduction in the dorsal raphe nucleus (Daniele et al., 2017; Moon et al., 2018). Additionally, when RSD was maintained for 5 days in mice, the monoamine oxidase A (MAOA) activity was potentiated in the amygdala and hippocampus, promoting the degradation of monoamine neurotransmitters, which may underlie depressive-like behaviors induced by RSD (Zhen et al., 2017). Interestingly in this work, and supporting our observations, RSD mice decreased sucrose preference rate.

Depression-like behaviors are related to increased peripheral blood levels of IL-1β, IL-6, TNF, and CRP, the most reliable biomarkers of inflammation (Miller and Raison, 2016). Thus, the anhedonia behavior observed in our RSD model (Figure 6B) could be a consequence of the inflammatory state triggered by RSD (by rising IL-1β and TNF-α levels in serum) (Figures 3E,F). A valid perspective could be to evaluate neurotransmitter levels in our model. IL-1 produces deficits in norepinephrine and 5-HT synthesis in several brain regions (Brebner et al., 2000; Dunn, 2006). On the other hand, TNF-α influences the central monoamine synthesis pathway, norepinephrine and 5-HT utilization rises in the paraventricular nucleus (PVN), the central amygdala, and the prefrontal cortex (Hayley et al., 1999; Brebner et al., 2000).

Moreover, IL-1β and TNF-α induction of p38 mitogen-activated protein kinase (MAPK) has been shown to increase the 5-HT reuptake, leading to lower synaptic availability and animals exhibit depressive-like behavior (Felger et al., 2013). In our study, QUIN administration ameliorated the anhedonia effect of RSD. Considering previous reports, this may be caused by downregulating IL-1β and TNF-α evoked by QUIN treatment. As mentioned above, it would be interesting to evaluate the levels of monoamine neurotransmitters to determine whether there is a neurochemical link between neuroinflammation and improving these depressive-like behaviors.

Alternatively, QUIN cognitive rescue could be through an increase in neuronal activity; however, the modulatory effects of D2DR are debatable. The influence of DA on glutamatergic transmission has been broadly explored, nevertheless is complex depending on the activated receptor. D2DR reduces the DA release since it is also pre-synaptic, and its activation decreases neuronal excitability (Kebabian and Greengard, 1971), which implies that the activation of synaptic plasticity machinery is limited. Little is known about QUIN’s effects on memory tasks; its administration has been associated with hyper-locomotion in rats and is employed as an experimental model of obsessive-compulsive disorder for causing hyper-locomotor behavior (Servaes et al., 2016). Interestingly, D2DR KO mice also exhibit increased DA synthesis and release with hyper-locomotor behavior derived from reduced expression in midbrain pre-synapses (Bello et al., 2011). Chronically QUIN administration has been shown to reverse spatial learning in rats, a task dependent on hippocampus integrity (Hatalova et al., 2014). These antecedents make it challenging to establish the frontier between locomotor and cognitive impairments in these models. Based on our results, it seems plausible that D2DR-dependent memory rescue depends on an additional stimulus, in this context, the inflammation derived from RSD.

Summarizing, our results show a potential beneficial role of D2DR in regulating the inflammatory response in RSD. We propose that the QUIN effect in ameliorating the behavioral impairments reported here is due to the anti-inflammatory effect regulated by the D2DR. Hence, this receptor may function as an interplay between inflammation and behavior when the system is challenged by an adverse stimulus triggered by REM sleep deprivation. However, the complexity of DA regulation in the immune system depends as well on other subtypes of DR, not only D2DR. Thus, in future studies, using a D2DR antagonist and D1-type receptor agonist, or vice versa, may be helpful to elucidate the immunomodulatory mechanisms’ activity of D2DR and how they influence behavioral impairments. Due to the complex regulatory network of neuroinflammation during RSD, the interaction of other functional systems like the dopaminergic system in their regulation should also be considered to develop new therapeutic strategies.

## Conclusion

Finally, our findings showed crosstalk between neuroinflammation (as evidenced by increased IL-1β and TNF-α levels) and D2DR in an RSD model. Behavioral tests revealed that systemic QUIN administration exerted a central effect, as evidenced by enhanced memory and sucrose preference compared to RSD. Notably, some cellular events, such as the microglial state, remain to be elucidated, which is crucial for responding to neuroinflammatory stimuli.

## Data availability statement

The raw data supporting the conclusions of this article will be made available by the authors, without undue reservation.

## Ethics statement

The animal study was reviewed and approved by Comité Interno para el Cuidado y Uso de Animales de Laboratorio (CICUAL), Facultad de Medicina, UNAM.

## Author contributions

All authors listed have made a substantial, direct, and intellectual contribution to the work, and approved it for publication.

## Abbreviations

5-HT: serotonin
BBB: blood–brain barrier
CNS: central nervous system
COX-2: cyclooxygenase-2
CRP: C-reactive protein
D2DR: dopamine D2 receptor
DA: dopamine
ET-1: endothelin-1
FO: familiar object
GPCR: G protein-coupled receptor
IFN γ: interferon-gamma
IGF-1: insulin-like growth factor 1
IL-1 β: interleukin 1 β
IL-6: interleukin 6
IL-10: interleukin 10
IL-12: interleukin 12
IL-17A: interleukin 17A
iNOS: inducible nitric oxide synthase
LPS: lipopolysaccharide
LTD: long-term depression
LTP: long-term potentiation
MAOA: monoamine oxidase A
MAPK: mitogen-activated protein kinase
MWM: Morris water maze
NO: novel object
NOR: novel object recognition
NREM: without rapid eye movement or non-REM
PP2A: phosphoprotein phosphatase 2
PVN: paraventricular nucleus
QUIN: quinpirole
REM: rapid eye movement
RI: recognition index
SEM: standard errors of the means
TE: total time spent exploring both objects
TH: tyrosine hydroxylase
TNF-α: tumor necrosis factor- α
TNO: time spent exploring the novel object
VEGF: vascular endothelial growth factor.

## References

[B1] Abel T. Havekes R. Saletin J. M. Walker M. P. (2013). Sleep, plasticity and memory from molecules to whole-brain networks. *Curr. Biol.* 23 R774–R788. 10.1016/j.cub.2013.07.025 24028961 PMC4263505

[B2] Alam S. I. Jo M. G. Park T. J. Ullah R. Ahmad S. Rehman S. U. (2021). Quinpirole-Mediated Regulation of Dopamine D2 Receptors Inhibits Glial Cell-Induced Neuroinflammation in Cortex and Striatum after Brain Injury. *Biomedicines* 9:47. 10.3390/biomedicines9010047 33430188 PMC7825629

[B3] Ali R. A. Qureshi M. A. McCorkle F. M. (1994). Profile of chicken macrophage functions after exposure to catecholamines in vitro. *Immunopharmacol. Immunotoxicol.* 16 611–625. 10.3109/08923979409019742 7876464

[B4] Antunes M. Biala G. (2012). The novel object recognition memory: Neurobiology, test procedure, and its modifications. *Cogn. Process.* 13 93–110. 10.1007/s10339-011-0430-z 22160349 PMC3332351

[B5] Ashley N. T. Sams D. W. Brown A. C. Dumaine J. E. (2016). Novel environment influences the effect of paradoxical sleep deprivation upon brain and peripheral cytokine gene expression. *Neurosci. Lett.* 615 55–59. 10.1016/j.neulet.2016.01.013 26806035 PMC4755797

[B6] Babson K. A. Trainor C. D. Feldner M. T. Blumenthal H. (2010). A test of the effects of acute sleep deprivation on general and specific self-reported anxiety and depressive symptoms: An experimental extension. *J. Behav. Ther. Exp. Psychiatry* 41 297–303. 10.1016/j.jbtep.2010.02.008 20231014 PMC2862829

[B7] Beaulieu J. M. Gainetdinov R. R. Caron M. G. (2007). The Akt-GSK-3 signaling cascade in the actions of dopamine. *Trends Pharmacol. Sci.* 28 166–172. 10.1016/j.tips.2007.02.006 17349698

[B8] Bello E. P. Mateo Y. Gelman D. M. Noaín D. Shin J. H. Low M. J. (2011). Cocaine supersensitivity and enhanced motivation for reward in mice lacking dopamine D2 autoreceptors. *Nat. Neurosci.* 14 1033–1038. 10.1038/nn.2862 21743470 PMC3175737

[B9] Bessa J. M. Mesquita A. R. Oliveira M. (2009). A transdimensional approach to the behavioral aspects of depression. *Front. Behav. Neurosci.* 3:1. 10.3389/neuro.08.001.2009 19194528 PMC2634526

[B10] Bevins R. A. Besheer J. (2006). Object recognition in rats and mice: A one-trial non-matching-to-sample learning task to study “recognition memory. *Nat. Protocols* 1 1306–1311. 10.1038/nprot.2006.205 17406415

[B11] Bourgognon J.-M. Cavanagh J. (2020). The role of cytokines in modulating learning and memory and brain plasticity. *Brain Neurosci. Adv.* 4:239821282097980. 10.1177/2398212820979802 33415308 PMC7750764

[B12] Brebner K. Hayley S. Zacharko R. (2000). Synergistic Effects of Interleukin-1β, Interleukin-6, and Tumor Necrosis Factor-α Central Monoamine, Corticosterone, and Behavioral Variations. *Neuropsychopharmacology* 22 566–580. 10.1016/S0893-133X(99)00166-9 10788757

[B13] Broadbent N. J. Gaskin S. Squire L. R. Clark R. E. (2010). Object recognition memory and the rodent hippocampus. *Learn. Mem.* 17 5–11. 10.1101/lm.1650110 20028732 PMC2807177

[B14] Butler M. P. O’Connor J. J. Moynagh P. N. (2004). Dissection of tumor-necrosis factor-α inhibition of long-term potentiation (LTP) reveals a p38 mitogen-activated protein kinase-dependent mechanism which maps to early—but not late—phase LTP. *Neuroscience* 124 319–326. 10.1016/j.neuroscience.2003.11.040 14980382

[B15] Cao J. Herman A. B. West G. B. Poe G. Savage V. M. (2020). Unraveling why we sleep: Quantitative analysis reveals abrupt transition from neural reorganization to repair in early development. *Sci. Adv.* 6:eaba0398. 10.1126/sciadv.aba0398 32948580 PMC7500925

[B16] Carey R. J. Damianopoulos E. N. Shanahan A. B. (2008). Cocaine effects on behavioral responding to a novel object placed in a familiar environment. *Pharmacol. Biochem. Behav.* 88 265–271. 10.1016/j.pbb.2007.08.010 17897705

[B17] Chang J. Y. Liu L.-Z. (2000). Catecholamines inhibit microglial nitric oxide production. *Brain Res. Bull.* 52 525–530. 10.1016/S0361-9230(00)00291-4 10974492

[B18] Charuchinda C. Supavilai P. Karobath M. Palacios J. M. (1987). Dopamine D2 receptors in the rat brain: Autoradiographic visualization using a high-affinity selective agonist ligand. *J. Neurosci.* 7 1352–1360. 10.1523/jneurosci.07-05-01352.1987 2437261 PMC6568828

[B19] Cohen S. J. Stackman R. W. (2015). Assessing rodent hippocampal involvement in the novel object recognition task. A review. *Behav. Brain Res.* 285 105–117. 10.1016/j.bbr.2014.08.002 25169255 PMC7008635

[B20] Colavito V. Fabene P. F. Grassi-Zucconi G. Pifferi F. Lamberty Y. Bentivoglio M. (2013). Experimental sleep deprivation as a tool to test memory deficits in rodents. *Front. Syst. Neurosci.* 7:106. 10.3389/fnsys.2013.00106 24379759 PMC3861693

[B21] Daniele T. M. da C. de Bruin P. F. C. Rios E. R. V. de Bruin V. M. S. (2017). Effects of exercise on depressive behavior and striatal levels of norepinephrine, serotonin and their metabolites in sleep-deprived mice. *Behav. Brain Res.* 332 16–22. 10.1016/j.bbr.2017.05.062 28572056

[B22] Dunn A. J. (2006). Effects of cytokines and infections on brain neurochemistry. *Clin. Neurosci. Res.* 6 52–68. 10.1016/j.cnr.2006.04.002 18079991 PMC2136407

[B23] Färber K. Pannasch U. Kettenmann H. (2005). Dopamine and noradrenaline control distinct functions in rodent microglial cells. *Mol. Cell. Neurosci.* 29 128–138. 10.1016/j.mcn.2005.01.003 15866053

[B24] Felger J. C. Li L. Marvar P. J. Woolwine B. J. Harrison D. G. Raison C. L. (2013). Tyrosine metabolism during interferon-alpha administration: Association with fatigue and CSF dopamine concentrations. *Brain Behav. Immun.* 31 153–160. 10.1016/j.bbi.2012.10.010 23072726 PMC3578984

[B25] Gonzalez P. V. Schiöth H. B. Lasaga M. Scimonelli T. N. (2009). Memory impairment induced by IL-1β is reversed by α-MSH through central melanocortin-4 receptors. *Brain Behav. Immun.* 23 817–822. 10.1016/j.bbi.2009.03.001 19275930

[B26] Goshen I. Kreisel T. Ben-Menachem-Zidon O. Licht T. Weidenfeld J. Ben-Hur T. (2008). Brain interleukin-1 mediates chronic stress-induced depression in mice via adrenocortical activation and hippocampal neurogenesis suppression. *Mol. Psychiatry* 13 717–728. 10.1038/sj.mp.4002055 17700577

[B27] Goshen I. Kreisel T. Ounallah-Saad H. Renbaum P. Zalzstein Y. Ben-Hur T. (2007). A dual role for interleukin-1 in hippocampal-dependent memory processes. *Psychoneuroendocrinology* 32 1106–1115. 10.1016/j.psyneuen.2007.09.004 17976923

[B28] Gulyás M. Bencsik N. Pusztai S. Liliom H. Schlett K. (2016). AnimalTracker: An ImageJ-Based Tracking API to Create a Customized Behaviour Analyser Program. *Neuroinformatics* 14 479–481. 10.1007/s12021-016-9303-z 27166960

[B29] Han X. Ni J. Wu Z. Wu J. Li B. Ye X. (2020). Myeloid-specific dopamine D2 receptor signalling controls inflammation in acute pancreatitis via inhibiting M1 macrophage. *Br. J. Pharmacol.* 177 2991–3008. 10.1111/bph.15026 32060901 PMC7280007

[B30] Haskó G. Szabó C. Merkel K. Bencsics A. Zingareli B. Kvetan V. (1996). Modulation of lipopolysaccharide-induced tumor necrosis factor-α and nitric oxide production by dopamine receptor agonists and antagonists in mice. *Immunol. Lett.* 49 143–147. 10.1016/0165-2478(96)02494-7 8739308

[B31] Hatalova H. Radostova D. Pistikova A. Vales K. Stuchlik A. (2014). Spatial reversal learning in chronically sensitized rats and in undrugged sensitized rats with dopamine d2-like receptor agonist quinpirole. *Front. Behav. Neurosci.* 8:122. 10.3389/fnbeh.2014.00122 24782730 PMC3990106

[B32] Hayley S. Brebner K. Lacosta S. Merali Z. Anisman H. (1999). Sensitization to the effects of tumor necrosis factor-α: Neuroendocrine, central monoamine, and behavioral variations. *J. Neurosci.* 19 5654–5665. 10.1523/JNEUROSCI.19-13-05654.1999 10377371 PMC6782322

[B33] He J. Hsuchou H. He Y. Kastin A. J. Wang Y. Pan W. (2014). Sleep restriction impairs blood-brain barrier function. *J. Neurosci.* 34 14697–14706. 10.1523/JNEUROSCI.2111-14.2014 25355222 PMC4212067

[B34] Hou J. Shen Q. Wan X. Zhao B. Wu Y. Xia Z. (2019). REM sleep deprivation-induced circadian clock gene abnormalities participate in hippocampal-dependent memory impairment by enhancing inflammation in rats undergoing sevoflurane inhalation. *Behav. Brain Res.* 364 167–176. 10.1016/j.bbr.2019.01.038 30779975

[B35] Hurtado-Alvarado G. Domínguez-Salazar E. Velázquez-Moctezuma J. Gómez-González B. (2016). A2A Adenosine Receptor antagonism reverts the blood-brain barrier dysfunction induced by sleep restriction. *PLoS One* 11:e0167236. 10.1371/journal.pone.0167236 27893847 PMC5125701

[B36] Ibrahim L. Duncan W. Luckenbaugh D. A. Yuan P. Machado-Vieira R. Zarate C. A. (2011). Rapid antidepressant changes with sleep deprivation in major depressive disorder are associated with changes in vascular endothelial growth factor (VEGF): A pilot study. *Brain Res. Bull.* 86 129–133. 10.1016/j.brainresbull.2011.06.003 21704134 PMC3156364

[B37] Kadomoto S. Izumi K. Mizokami A. (2022). Macrophage polarity and disease control. . *Int. J. Mol. Sci.* 23:144. 10.3390/ijms23010144 35008577 PMC8745226

[B38] Kebabian J. W. Greengard P. (1971). Dopamine-sensitive adenyl cyclase: Possible role in synaptic transmission. *Science* 174 1346–1349. 10.1126/science.174.4016.1346 4332627

[B39] Killgore W. D. S. (2010). Effects of sleep deprivation on cognition. *Prog. Brain Res.* 185 105–129. 10.1016/B978-0-444-53702-7.00007-5 21075236

[B40] Kincheski G. C. Valentim I. S. Clarke J. R. Cozachenco D. Castelo-Branco M. T. L. Ramos-Lobo A. M. (2017). Chronic sleep restriction promotes brain inflammation and synapse loss and potentiates memory impairment induced by amyloid-β oligomers in mice. *Brain Behav. Immun.* 64 140–151. 10.1016/j.bbi.2017.04.007 28412140

[B41] Krueger J. M. Frank M. G. Wisor J. P. Roy S. (2016). Sleep function: Toward elucidating an enigma. *Sleep Med. Rev.* 28 46–54. 10.1016/j.smrv.2015.08.005 26447948 PMC4769986

[B42] Kuo D. Y. (2002). Co-administration of dopamine D1 and D2 agonists additively decreases daily food intake, body weight and hypothalamic neuropeptide Y level in rats. *J. Biomed. Sci.* 9 126–132. 10.1159/000048208 11914579

[B43] Kushida C. A. (2016). *Sleep deprivation: Basic science, physiology and behavior.* Boca Raton: CRC Press.

[B44] Labrousse V. F. Costes L. Aubert A. Darnaudéry M. Ferreira G. Amédée T. (2009). Impaired Interleukin-1β and c-Fos Expression in the hippocampus is associated with a spatial memory deficit in P2X7 receptor-deficient mice. *PLoS One* 4:e6006. 10.1371/journal.pone.0006006 19547756 PMC2695542

[B45] Lee Y.-D. Kim J.-Y. Lee K.-H. Kwak Y.-J. Lee S.-K. Kim O.-S. (2009). Melatonin attenuates lipopolysaccharide-induced acute lung inflammation in sleep-deprived mice. *J. Pineal Res.* 46 53–57. 10.1111/j.1600-079X.2008.00621.x 18673421

[B46] Levey A. I. Hersch S. M. Rye D. B. Sunahara R. K. Niznik H. B. Kitt C. A. (1993). Localization of D1 and D2 dopamine receptors in brain with subtype-specific antibodies. *Proc. Natl. Acad. Sci. U.S.A.* 90 8861–8865. 10.1073/pnas.90.19.8861 8415621 PMC47460

[B47] Li M. Zhou L. Sun X. Yang Y. Zhang C. Wang T. (2022). Dopamine, a co-regulatory component, bridges the central nervous system and the immune system. *Biomed. Pharmacother.* 145:112458. 10.1016/j.biopha.2021.112458 34847478

[B48] Li S. Tian Y. Ding Y. Jin X. Yan C. Shen X. (2009). The effects of rapid eye movement sleep dep rivation and recovery on spatial reference memory of young rats. *Learn. Behav.* 37 246–253. 10.3758/LB.37.3.246 19542091

[B49] Liggins J. (2009). The roles of dopamine D1 and D2 receptors in working memory function. *McGill Sci. Undergrad. Res. J.* 4 39–45.

[B50] Liu M. Y. Yin C. Y. Zhu L. J. Zhu X. H. Xu C. Luo C. X. (2018). Sucrose preference test for measurement of stress-induced anhedonia in mice. *Nat. Protocols* 13 1686–1698. 10.1038/s41596-018-0011-z 29988104

[B51] Liu P. Qin D. Lv H. Fan W. Zhou F. Gao Z. (2021). Activation of dopamine D2 receptor alleviates neuroinflammation in a mouse model of allergic rhinitis with olfactory dysfunction. *Allergy Asthma Immunol. Res.* 13:882. 10.4168/aair.2021.13.6.882 34734506 PMC8569020

[B52] Lueptow L. M. (2017). Novel object recognition test for the investigation of learning and memory in mice. *J. Vis. Exp.* 126:55718. 10.3791/55718 28892027 PMC5614391

[B53] Machado R. B. Hipólide D. C. Benedito-Silva A. A. Tufik S. (2004). Sleep deprivation induced by the modified multiple platform technique: Quantification of sleep loss and recovery. *Brain Res.* 1004 45–51. 10.1016/j.brainres.2004.01.019 15033418

[B54] Matt S. M. Gaskill P. J. (2020). Where Is dopamine and how do immune cells see it?: Dopamine-mediated immune cell function in health and disease. *J. Neuroimmune. Pharmacol.* 15 114–164. 10.1007/s11481-019-09851-4 31077015 PMC6842680

[B55] McCoy J. G. Strecker R. E. (2011). The cognitive cost of sleep lost. *Neurobiol. Learn. Mem.* 96 564–582. 10.1016/j.nlm.2011.07.004 21875679 PMC3614362

[B56] McCoy J. G. Christie M. A. Kim Y. Brennan R. Poeta D. L. McCarley R. W. (2013). Chronic sleep restriction impairs spatial memory in rats. *NeuroReport* 24 91–95. 10.1097/WNR.0b013e32835cd97a 23238166 PMC3620033

[B57] Mikulak J. Bozzo L. Roberto A. Pontarini E. Tentorio P. Hudspeth K. (2014). Dopamine Inhibits the Effector Functions of Activated NK Cells via the Upregulation of the D5 Receptor. *J. Immunol.* 193 2792–2800. 10.4049/JIMMUNOL.1401114/-/DCSUPPLEMENTAL 25127864

[B58] Miller A. H. Raison C. L. (2016). The role of inflammation in depression: From evolutionary imperative to modern treatment target. *Nat. Rev. Immunol.* 16 22–34. 10.1038/nri.2015.5 26711676 PMC5542678

[B59] Moon E. J. Ko I. G. Kim S. E. Jin J. J. Hwang L. Kim C. J. (2018). Dexmedetomidine ameliorates sleep deprivation-induced depressive behaviors in mice. *Int. Neurourol. J.* 22 S139–S146. 10.5213/inj.1836228.114 30396263 PMC6234724

[B60] Murakami T. Enjoji M. Koyama S. (2018). Leptin attenuates D 2 receptor-mediated inhibition of putative ventral tegmental area dopaminergic neurons. *Physiol. Rep.* 6:e13631. 10.14814/phy2.13631 29611323 PMC5880875

[B61] Nyberg L. Karalija N. Salami A. Andersson M. Wåhlin A. Kaboovand N. (2016). Dopamine D2 receptor availability is linked to hippocampal–caudate functional connectivity and episodic memory. *Proc. Natl. Acad. Sci. U.S.A.* 113 7918–7923. 10.1073/pnas.1606309113 27339132 PMC4948341

[B62] Péczely L. Ollmann T. László K. Kovács A. Gálosi R. Kertes E. (2016). Role of ventral pallidal D2 dopamine receptors in the consolidation of spatial memory. *Behav. Brain Res.* 313 1–9. 10.1016/j.bbr.2016.07.007 27392640

[B63] Peiser C. Trevisani M. Groneberg D. A. Dinh Q. T. Lencer D. Amadesi S. (2005). Dopamine type 2 receptor expression and function in rodent sensory neurons projecting to the airways. *Am. J. Physiol. Lung Cell. Mol. Physiol.* 289 L153–L158. 10.1152/ajplung.00222.2004 15792966

[B64] Piber D. (2021). The role of sleep disturbance and inflammation for spatial memory. *Brain Behav. Immun. Health* 17:100333. 10.1016/j.bbih.2021.100333 34589818 PMC8474561

[B65] Pinoli M. Marino F. Cosentino M. (2017). Dopaminergic regulation of innate immunity: A Review. *J. Neuroimmune. Pharmacol.* 12 602–623. 10.1007/s11481-017-9749-2 28578466

[B66] Porkka-Heiskanen T. Zitting K.-M. Wigren H.-K. (2013). Sleep, its regulation and possible mechanisms of sleep disturbances. *Acta Physiologica* 208 311–328. 10.1111/apha.12134 23746394

[B67] Reger M. L. Hovda D. A. Giza C. C. (2009). Ontogeny of rat recognition memory measured by the novel object recognition task. *Dev. Psychobiol.* 51 672–678. 10.1002/dev.20402 19739136 PMC2956740

[B68] Rocchetti J. Isingrini E. Dal Bo G. Sagheby S. Menegaux A. Tronche F. (2015). Presynaptic D2 dopamine receptors control long-term depression expression and memory processes in the temporal hippocampus. *Biol. Psychiatry* 77 513–525. 10.1016/j.biopsych.2014.03.013 24742619

[B69] Roy B. Rai U. (2004). Dual mode of catecholamine action on splenic macrophage phagocytosis in wall lizard. Hemidactylus flaviviridis. *Gen. Comp. Endocrinol.* 136 180–191. 10.1016/j.ygcen.2003.12.023 15028521

[B70] Ruiz F. S. Andersen M. L. Martins R. C. Zager A. Lopes J. D. Tufik S. (2012). Immune alterations after selective rapid eye movement or total sleep deprivation in healthy male volunteers. *Innate Immun.* 18 44–54. 10.1177/1753425910385962 21088046

[B71] Sarkar C. Basu B. Chakroborty D. Dasgupta P. S. Basu S. (2010). The immunoregulatory role of dopamine: An update. *Brain Behav. Immun.* 24 525–528. 10.1016/j.bbi.2009.10.015 19896530 PMC2856781

[B72] Scheggi S. de Montis M. G. Gambarana C. (2018). Making sense of rodent models of anhedonia. *Int. J. Neuropsychopharmacol.* 21 1049–1065. 10.1093/ijnp/pyy083 30239762 PMC6209858

[B73] Servaes S. Glorie D. Verhaeghe J. wyffels L. Stroobants S. Staelens S. (2016). [18F]-FDG PET neuroimaging in rats with quinpirole-induced checking behavior as a model for obsessive compulsive disorder. *Psychiatry Res. Neuroimaging* 257 31–38. 10.1016/j.pscychresns.2016.10.003 27771554

[B74] Shin J.-W. Cheong Y.-J. Koo Y.-M. Kim S. Noh C.-K. Son Y.-H. (2014). α-Asarone ameliorates memory deficit in lipopolysaccharide-treated mice via suppression of pro-inflammatory cytokines and microglial activation. *Biomole. Ther.* 22 17–26. 10.4062/biomolther.2013.102 24596617 PMC3936426

[B75] Singh A. Jones O. D. Mockett B. G. Ohline S. M. Abraham W. C. (2019). Tumor necrosis factor-α-mediated metaplastic inhibition of LTP Is constitutively engaged in an Alzheimer’s Disease model. *J. Neurosci.* 39 9083–9097. 10.1523/JNEUROSCI.1492-19.2019 31570539 PMC6855674

[B76] Sobrian S. K. Marr L. Ressman K. (2003). Prenatal cocaine and/or nicotine exposure produces depression and anxiety in aging rats. *Prog. Neuro Psychopharmacol. Biol. Psychiatry* 27 501–518. 10.1016/S0278-5846(03)00042-3 12691787

[B77] Suchecki D. Lobo L. L. Hipólide D. C. Tufik S. (1998). Increased ACTH and corticosterone secretion induced by different methods of paradoxical sleep deprivation. *J. Sleep Res.* 7 276–281. 10.1046/j.1365-2869.1998.00122.x 9844854

[B78] Suresh K. Shankar V. Dayanand C. D. (2021). Impact of REM sleep deprivation and sleep recovery on circulatory neuroinflammatory markers. *Sleep Sci.* 14 64–68. 10.5935/1984-0063.20190157 34104339 PMC8157773

[B79] Taglialatela G. Hogan D. Zhang W. R. Dineley K. T. (2009). Intermediate- and long-term recognition memory deficits in Tg2576 mice are reversed with acute calcineurin inhibition. *Behav. Brain Res.* 200 95–99. 10.1016/j.bbr.2008.12.034 19162087 PMC2663011

[B80] Thomas Broome S. Louangaphay K. Keay K. A. Leggio G. M. Musumeci G. Castorina A. (2020). Dopamine: An immune transmitter. *Neural. Regen. Res.* 15 2173–2185. 10.4103/1673-5374.284976 32594028 PMC7749467

[B81] Vorhees C. v Williams M. T. (2006). Morris water maze: Procedures for assessing spatial and related forms of learning and memory. *Nat. Protocols* 1 848–858. 10.1038/nprot.2006.116 17406317 PMC2895266

[B82] Wadhwa M. Prabhakar A. Ray K. Roy K. Kumari P. Jha P. K. (2017). Inhibiting the microglia activation improves the spatial memory and adult neurogenesis in rat hippocampus during 48h of sleep deprivation. *J. Neuroinflam.* 14:222. 10.1186/s12974-017-0998-z 29141671 PMC5688670

[B83] Walker M. P. (2008). Sleep-dependent memory processing. *Harvard Rev. Psychiatry* 16 287–298. 10.1080/10673220802432517 18803104

[B84] Wang B. Chen T. Li G. Jia Y. Wang J. Xue L. (2019). Dopamine alters lipopolysaccharide-induced nitric oxide production in microglial cells via activation of D1-Like Receptors. *Neurochem. Res.* 44 947–958. 10.1007/S11064-019-02730-7 30659504

[B85] Ward C. P. Wooden J. I. Kieltyka R. (2017). Effects of sleep deprivation on spatial learning and memory in juvenile and young adult rats. *Psychol. Neurosci.* 10 109–116. 10.1037/pne0000075 28959381 PMC5612336

[B86] Willner P. Towell A. Sampson D. Sophokleous S. Muscat R. (1987). Reduction of sucrose preference by chronic unpredictable mild stress, and its restoration by a tricyclic antidepressant. *Psychopharmacology* 93 358–364. 10.1007/BF00187257 3124165

[B87] Xue R. Wan Y. Sun X. Zhang X. Gao W. Wu W. (2019). Nicotinic mitigation of neuroinflammation and oxidative stress after chronic sleep deprivation. *Front. Immunol.* 10:2546. 10.3389/fimmu.2019.02546 31736967 PMC6828928

[B88] Yang K. Broussard J. I. Levine A. T. Jenson D. Arenkiel B. R. Dani J. A. (2017). Dopamine receptor activity participates in hippocampal synaptic plasticity associated with novel object recognition. *Eur. J. Neurosci.* 45 138–146. 10.1111/ejn.13406 27646422 PMC5209255

[B89] Yin M. Chen Y. Zheng H. Pu T. Marshall C. Wu T. (2017). Assessment of mouse cognitive and anxiety-like behaviors and hippocampal inflammation following a repeated and intermittent paradoxical sleep deprivation procedure. *Behav. Brain Res.* 321 69–78. 10.1016/j.bbr.2016.12.034 28043900

[B90] Yue S. Wang T. Yang Y. Fan Y. Zhou L. Li M. (2021). Lipopolysaccharide/D-galactosamine-induced acute liver injury could be attenuated by dopamine receptor agonist rotigotine via regulating NF-κB signaling pathway. *Int. Immunopharmacol.* 96:107798. 10.1016/j.intimp.2021.107798 34162160

[B91] Zager A. Andersen M. L. Ruiz F. S. Antunes I. B. Tufik S. (2007). Effects of acute and chronic sleep loss on immune modulation of rats. *Am. J. Physiol. Regulatory Integr. Comp. Physiol.* 293 R504–R509. 10.1152/ajpregu.00105.2007 17409265

[B92] Zhang Y. Chen Y. Wu J. Manaenko A. Yang P. Tang J. (2015). Activation of Dopamine D2 receptor suppresses neuroinflammation through αB-Crystalline by Inhibition of NF-κB nuclear translocation in experimental ICH mice model. *Stroke* 46 2637–2646. 10.1161/STROKEAHA.115.009792 26251254 PMC4550518

[B93] Zhen W. Lu C. Ling Z. Xuemin W. (2017). Paradoxical sleep deprivation modulates depressive-like behaviors by regulating the MAOA levels in the amygdala and hippocampus. *Brain Res.* 1664 17–24. 10.1016/j.brainres.2017.03.022 28365314

[B94] Zhou Q. G. Hu Y. Wu D. L. Zhu L. J. Chen C. Jin X. (2011). Hippocampal telomerase is involved in the modulation of depressive behaviors. *J. Neurosci.* 31 12258–12269. 10.1523/JNEUROSCI.0805-11.2011 21865469 PMC6623221

[B95] Zielinski M. R. Kim Y. Karpova S. A. McCarley R. W. Strecker R. E. Gerashchenko D. (2014). Chronic sleep restriction elevates brain interleukin-1 beta and tumor necrosis factor-alpha and attenuates brain-derived neurotrophic factor expression. *Neurosci. Lett.* 580 27–31. 10.1016/j.neulet.2014.07.043 25093703 PMC4162816

